# Comment on Dijkstra et al. A False-Negative Newborn Screen for Tyrosinemia Type 1—Need for Re-Evaluation of Newborn Screening with Succinylacetone. *Int. J. Neonatal Screen.* 2023, *9*, 66

**DOI:** 10.3390/ijns10040065

**Published:** 2024-09-24

**Authors:** Marelle J. Bouva, Rose E. Maase, Ruurd M. van Elburg

**Affiliations:** Department Public Health Genomics & Screening, Centre for Health Protection, National Institute for Public Health and the Environment, 3720 BA Bilthoven, The Netherlands; marelle.bouva@rivm.nl (M.J.B.); ruurd.van.elburg@rivm.nl (R.M.v.E.)

The assessment of newborn screening (NBS) algorithms’ performance to ensure quality improvements is a continuous process: false-positive referrals can enable optimisations in the shorter term, but false-negative referrals are often only discovered many years after the screening has taken place. In their article ‘A False-Negative Newborn Screen for Tyrosinemia Type 1—Need for Re-Evaluation of Newborn Screening with Succinylacetone’ (*Int. J. Neonatal Screen.* **2023**, *9*, 66. https://doi.org/10.3390/ijns9040066), Dijkstra et al. report a 9-year-old patient with liver pathology caused by Tyrosinemia Type 1 (TT1) despite a negative NBS result [1]. The TT1 screening was based on the concentration of succinylacetone (SA), which is widely used as a screening marker for TT1. This case is the first reported false-negative newborn screening for TT1 to our knowledge. We compliment the authors for their report and recommendation that a possible false-negative NBS should be considered when a child presents with liver pathology. The case report illustrates the challenge of population-based NBS, which strives for a 100% sensitivity, a specificity that is as high as possible and the limitation of false-positive results.

The reported false-negative screening had an SA concentration of 1.08 μmol/L blood in a dried blood card drawn at day 4 after birth. The screening was performed with the PerkinElmer NeoBase assay on a Waters Quattro Micro using a cut-off value of 1.20 μmol/L blood. This cut-off value was established by the Dutch NBS programme and was based on the SA concentrations measured in dried blood spots received for the purpose of NBS.

TT1 NBS was implemented in The Netherlands in 2007 and, since October 2008, has been conducted using SA as screening marker. Between October 2008 and December 2021, 2,339,258 newborns were screened for TT1 in The Netherlands: 48 newborns were referred to a pediatrician, of which 37 were reported as a false-positive result. With this single false-negative result that has been reported by Dijkstra et al., the sensitivity of the TT1 screening for the entire period between October 2008 and December 2021 is 92%. 

In their introduction, the authors advocate for an evaluation of the NBS for TT1 based on the use of the SA marker. Such an extensive evaluation is currently being performed by the Reference Laboratory for NBS at the National Institute for Public Health and the Environment in The Netherlands. This evaluation is based on nationwide NBS data collected from 2008 to 2021 and aims to determine the most optimal way to screen for TT1, i.e., how to achieve the highest sensitivity with the lowest rate of false-positive patients. 

Since only one report of a missed TT1 case (from TT1 NBS based on SA) is known, we would urge caution in the adjustment of previously established cut-off values to improve the performance of TT1 screening, as suggested by Dijkstra et al. Following the transition to the NeoBase2 kit/Waters Xevo TQD (from NeoBase1/Waters Quattro Micro) in 2018 and the resulting lower limit of detection with the new kit/instrument, we reduced our SA cut-off value to 0.60 μmol/L. This was not related to screening optimalisation, as alluded to by the authors in their discussion. In fact, as part of the ongoing evaluation, the current cut-off value is being investigated: we are aware that our cut-off value is lower than other programmes using the NeoBase2 kit and that this may be the cause of our current high number of false-positive referrals. 

Because of the known lower cut-off value for SA in The Netherlands, our current programme may already be detecting the mild phenotypes. It would be interesting to establish whether there is a link between disease severity and the SA concentration through a long-term follow-up evaluation of thescreening, in collaboration with the clinicians. This kind of information might help further improve the performance of screening programmes. 

This false-negative case underscores the complexity associated with the establishment of NBS for rare diseases and the value of case reports for the continuous improvement of the entire screening process. 
